# Identification of tick-borne pathogens by metagenomic next-generation sequencing in *Dermacentor nuttalli* and *Ixodes persulcatus* in Inner Mongolia, China

**DOI:** 10.1186/s13071-021-04740-3

**Published:** 2021-05-27

**Authors:** Jun Jiao, Zhiyu Lu, Yonghui Yu, Yangxuan Ou, Mengjiao Fu, Yuee Zhao, Nier Wu, Mingliang Zhao, Yan Liu, Yi Sun, Bohai Wen, Dongsheng Zhou, Qinghong Yuan, Xiaolu Xiong

**Affiliations:** 1 State Key Laboratory of Pathogen and Biosecurity, Beijing Institute of Microbiology and Epidemiology, Beijing, PR China; 2 Yunnan Institute of Endemic Disease Control and Prevention, Yunnan Provincial Key Laboratory of Natural Focal Disease Control and Prevention, Yunnan, PR China; 3 Department of Microbiology, School of Basic Medical Sciences, Anhui Provincial Laboratory of Microbiology and Parasitology, Anhui Key Laboratory of Zoonoses, Anhui Medical University, Hefei, PR China

**Keywords:** *Dermacentor nuttalli*, *Ixodes persulcatus*, Spotted fever group *Rickettsia*, *Anaplasma* sp. Mongolia, *Coxiella-*like endosymbiont, *Babesia venatorum*, Inner Mongolia

## Abstract

**Background:**

Hard ticks act as arthropod vectors in the transmission of human and animal pathogens and are widely distributed in northern China. The aim of this study is to screen the important tick-borne pathogens (TBPs) carried by hard ticks in Inner Mongolia using metagenomic next-generation sequencing (mNGS) and to estimate the risk of human infection imposed by tick bites.

**Methods:**

The adult *Dermacentor nuttalli* (*n* = 203) and *Ixodes persulcatus* (*n* = 36) ticks feeding on cattle were collected. The pooled DNA samples prepared from these ticks were sequenced as the templates for mNGS to survey the presence of TBPs at the genus level. Individual tick DNA samples were detected by genus--specific or group-specific nested polymerase chain reaction (PCR) of these TBPs and combined with DNA sequencing assay to confirm the results of mNGS.

**Results:**

*R. raoultii* (45.32%, 92/203), *Candidatus R. tarasevichiae* (5.42%, 11/203), *Anaplasma* sp. Mongolia (26.60%, 54/203), *Coxiella-*like endosymbiont (CLE) (53.69%, 109/203), and *Babesia venatorum* (7.88%, 16/203) were detected in *D. nuttalli*, while *R. raoultii* (30.56%, 11/36), *Anaplasma* sp. Mongolia (27.80%, 10/36), and CLE (27.80%, 10/36) were detected in *I. persulcatus*. The double- and triple-pathogen/endosymbiont co-infections were detected in 40.39% of *D. nuttalli* and 13.89% of *I. persulcatus*, respectively. The dual co-infection with *R. raoultii* and CLE (14.29%, 29/203) and triple co-infection with *R. raoultii*, *Anaplasma* sp. Mongolia, and CLE (13.79%, 28/203) were most frequent in *D. nuttalli*.

**Conclusions:**

This study provides insight into the microbial diversity of *D. nuttalli* and *I. persulcatus* in Inner Mongolia, China, reporting for the first time that *Candidatus R. tarasevichiae* had been found in *D. nuttalli* in China, and for the first time in the world that *Anaplasma* sp. Mongolia has been detected in *I. persulcatus*. This study proves that various vertically transmitted pathogens co-inhabit *D. nuttalli* and *I. persulcatus*, and indicates that cattle in Inner Mongolia are exposed to several TBPs.

**Graphic Abstract:**

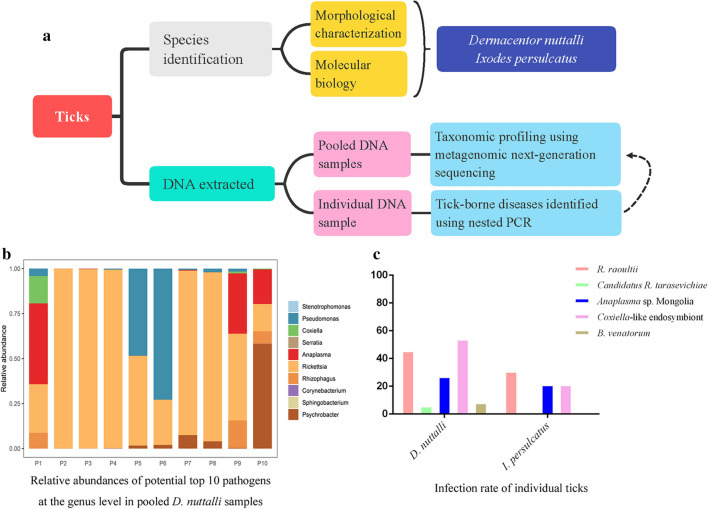

**Supplementary Information:**

The online version contains supplementary material available at 10.1186/s13071-021-04740-3.

## Background

Hard ticks (Acari: Ixodidae) are obligate blood-sucking parasitic arthropods which can infest mammals, birds, and reptiles, and act as arthropod vectors in the transmission of human and animal pathogens. A wide variety of pathogens can be maintained and transmitted by hard ticks, including *Ehrlichia* spp., *Anaplasma* spp., *Rickettsia* spp., *Coxiella* spp., *Babesia* spp., *Borrelia* spp., etc. [1]. In addition, a variety of endosymbionts, such as *Coxiella*-like, *Rickettsia*-like, and *Arsenophonus*-like endosymbionts, live inside hard ticks [2–4]. Therefore, hard ticks are usually considered to be the most important vectors of pathogens, and knowledge of the microbial communities within these ticks will be of benefit for risk assessment of tick-borne diseases.

China covers approximately 9.6 million square kilometers of land area. At least 117 tick species in ten genera of two families have been reported in China [5], with *Ixodes persulcatus*, *Dermacentor nuttalli*, *Hyalomma asiaticum*, *Dermacentor marginatus*, and *Dermacentor niveus* being the most common [6]. Increasing numbers of cases of human tick-borne diseases, including spotted fever [7], Q fever [8], anaplasmoses, ehriochioses [9], tick-borne encephalitis [10], and babesiosis [11], have been reported in China due to climate change combined with human movement into tick habitats. Therefore, human co-infection with more than one tick-borne pathogen (TBP) may occur after tick bite [12–14].

Mongolia Hulunbuir League, one of the important pastoral regions in Inner Mongolia in China, contains significant amounts of pastures and is an important region for animal production. The eastern part of Hulunbuir, stretching across the primeval forest-covered area in the Daxing'anling Mountains, is an important habitat for hard ticks [15]. Many TBPs including *C. burnetii* [16], *Rickettsia* spp. [16–18], *Anaplasma* spp. [19], and tick-borne encephalitis virus [20] have been detected in hard ticks collected here. However, little attention has been given to co-infection with TBPs in ticks, and continued research is needed to fully comprehend the diversity of TBPs. In this study, we investigated the microbial communities in hard ticks collected from cattle in Hulunbuir League to reveal the coexistence of TBPs using metagenomic next-generation sequencing (mNGS) combined with nested polymerase chain reaction (PCR). The results of our study might provide broader knowledge of the microorganisms inside hard ticks in the region, thereby strengthening programs to prevent and control the potential infections caused by TBPs.

## Methods

### Collection and identification of ticks

**Fig. 1:**
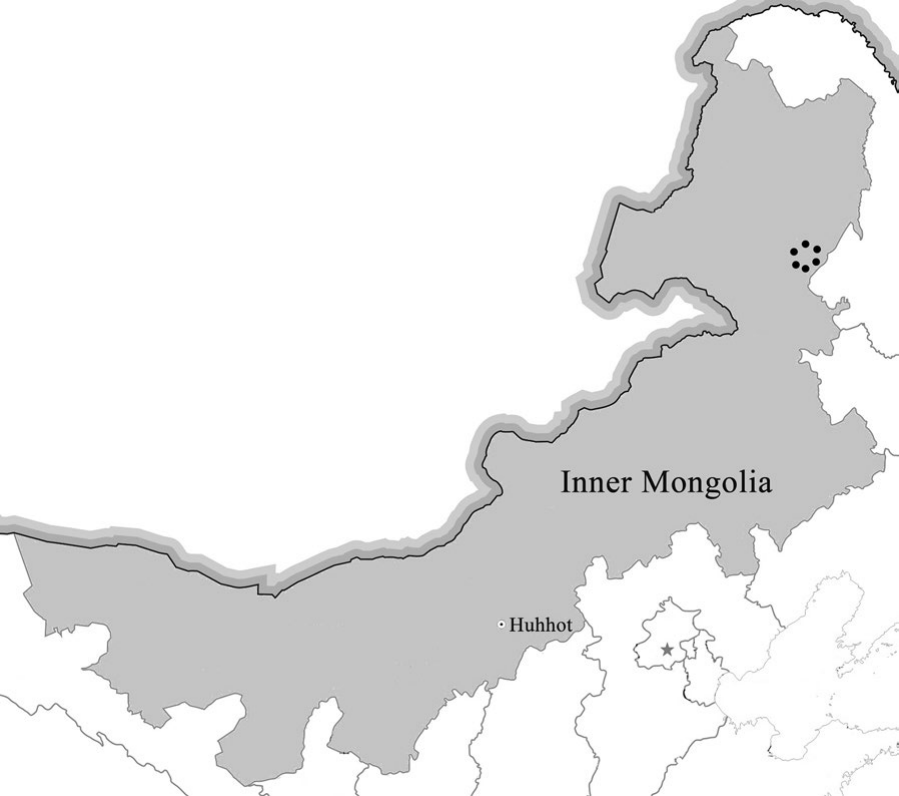
Map of the sampling sites in Inner Mongolia, China. The black dots indicate the sampling regions in this study

All ticks collected were feeding on cattle in Balin Town (E 122°24′10″, N 48°19′26″; E 122°22′12″, N 48°20′11″; E 122°22′13″, N 48°19′47″; E 122°24′59″, N 48°19′55″; E 122°21′11″, N 48°19′59″; E 122°20′59″, N 48°19′16″), Yake City, Hulunbuir League, Inner Mongolia, from April to October in 2019 (Fig. 1). Tick species were identified based on morphological characterization and by molecular biology methods based on the sequences of species-specific *16S rRNA* and mitochondrial cytochrome c oxidase I (COI) genes as previously described [21]. Following identification, the ticks were stored at −80 °C for further analysis.

### Tick washing, homogenization, and DNA extraction

To remove environmental contaminants, each tick was surface-sterilized twice with 75% ethanol, followed by phosphate-buffered saline (PBS) twice. Ticks were then individually homogenized in 300 μL of PBS using MagNA Lyser Green Beads (Roche, Mannheim, Germany), and DNA extraction was performed on 200 μL of each tick homogenate using a QIAamp^®^ Fast DNA Tissue Kit (Qiagen, Dusseldorf, Germany) according to the manufacturer’s instructions. The extracted genomic DNA was dissolved in 100 μL ultrapure water and stored at −20 °C for further analysis. For these previous steps, ultrapure water, sterile tubes, and filter tips were used, and all operations were carried out in a biological safety cabinet. Each time DNA extraction was performed, an extraction control (water) was added.

Individual DNA samples were mixed in an equal volume (20 μL) to prepare pooled DNA samples for full microbial genome sequencing using mNGS.

### Metagenome assembly, gene prediction, and taxonomy prediction

All pooled DNA samples were paired-end sequenced on the Illumina HiSeq platform (insert size 350 bp, read length 150 bp) by the Beijing Genomics Institute (BGI) (Beijing, China). The reads with more than 40 nt low-quality bases (quality value ≤ 38) were removed. Meanwhile, the reads with more than 10 nt “N” bases were filtered out of the data sets. Lastly, the reads overlapping more than 15 nt bases with the adapters were removed. Reads that aligned to tick genes were also removed using Bowtie 2 (v2.2.4) with the parameters -end-to-end, -sensitive, -I 200, -X 400 [22, 23]. Accordingly, the clean data were obtained.

Then the clean reads were mapped against scaffolds using SOAPdenovo (V2.04) with the parameters -d 1, -M 3, -R, -u, -F, -K 55 [24]. The unused reads from each sample were then assembled using the same parameters. The scaffolds were broken at N into the scaftigs [25], and the scaftigs with the length of ≥ 500 nt were used for further analysis [26]. Open reading frames (ORFs) in the scaftigs (≥ 500 bp) were predicted by MetaGeneMark (V2.10) [23, 27]. A nonredundant gene catalog was obtained after processing by using CD-HIT (V4.5.8) with the parameters -c 0.95, -G 0, -aS 0.9, -g 1, -d 0 [28, 29], and using a sequence identity threshold of 0.95 and a minimum coverage cutoff of 0.9. To determine the gene abundances, the reads were realigned with the gene catalog using Bowtie 2 and the following parameters: -m 200 -× 400 -s 119. Only genes with ≥ 2 mapped reads were deemed to be present in a sample [30]. Relative abundance of genes was calculated based on the number of reads mapped to the genes and the length of the genes as previously described [31–33].

To access the taxonomic assignments of genes, genes were aligned to the integrated NR database (version: 2018-01-02) of NCBI using DIAMOND (V0.9.9) and default parameters, with the exception of -k 50 -sensitive -e 0.00001 [34]. For each gene, the significant matches which were defined by e-values ≤ 10 * e-value of the top hit were retained to distinguish taxonomic groups [30]. Then the taxonomical level of each gene was determined by using the lowest common ancestor (LCA)-based algorithm implemented in MEGAN [35]. The results containing the number of genes and the abundance information for each sample, and the relative abundances of each taxonomic group were calculated by adding the relative abundances of genes annotated to the same feature [23, 26, 36].

### Polymerase chain reaction (PCR)

Based on the results of mNGS, genus-/group-specific PCR was performed to confirm the presence of TBPs in individual ticks. PCR was performed using a PCR System 9700 (Applied Biosystems, GeneAmp^®^, USA). For nested PCR, 1 μL of each individual DNA sample (150–330 ng) was used as template for the first round, and 1 μL of the primary PCR production was used as template for the second round. For the first round, a negative control (water) and an extraction control mentioned above were included in each PCR experiment. Tube strips with individual caps were used in amplification steps to prevent cross-contamination, and all PCR amplifications were carried out using PrimeSTAR^®^ HS (Premix) (TaKaRa, Beijing, China). All operations were carried out in a biological safety cabinet. Amplified products were then electrophoresed in 1.5% agarose gel, and the positive amplicons were sent to TSINGKE Biological Technology (Beijing, China) for sequencing. The PCR primers for the spotted fever group *Rickettsia* (SFGR) [37], *Anaplasma* spp. and *Ehrlichia* spp. [38], *Coxiella* spp. [39], and *Babesia* spp. [40] are presented in Table 1.

**Table 1:** Target genes and primer sequences used for nested PCR

Pathogen	Target gene	Primer name	Sequence (5′–3′)	Tm (T/℃)
SFGR	*gltA*	CS2d	ATGACCAATGAAAATAATAAT	50
		CSEndr	CTTATACTCTCTATGTACA	
		RpCS.877p	GGGGACCTGCTCACGGCGG	48
		RpCS.1258n	ATTGCAAAAAGTACAGTGAACA	
*Anaplasma* spp.	*16S rRNA*	Eh-out1	TTGAGAGTTTGATCCTGGCTCAGAACG	55
*Ehrlichia* spp.		Eh-out2	CACCTCTACACTAGGAATTCCGCTATC	
		Eh-gs1	GTAATAACTGTATAATCCCTG	55
		Eh-gs2	GTACCGTCATTATCTTCCCTA	
*Coxiella* spp.	*16S rRNA*	Cox16SF1	CGTAGGAATCTACCTTRTAGWGG	55
		Cox16SR2	GCCTACCCGCTTCTGGTACAATT	
		Cox16SF1	CGTAGGAATCTACCTTRTAGWGG	55
		Cox16SR1	ACTYYCCAACAGCTAGTTCTCA	
*Babesia* spp.	*18S rRNA*	Piro0F	GCCAGTAGTCATATGCTTGTGTTA	56
		Piro6R	CTCCTTCCTYTAAGTGATAAGGTTCAC	
		Piro1F	CCATGCATGTCTWAGTAYAARCTTTTA	56
		Piro5.5R	CCTYTAAGTGATAAGGTTCACAAAACTT	

### Phylogenetic analysis

The obtained DNA sequences were compared with those available in GenBank using the National Center for Biotechnology Information (NCBI; Bethesda, MD) Basic Local Alignment Search Tool (BLAST) search engine (http://blast.ncbi.nlm.nih.gov/blast.cgi), and multiple sequence alignment was performed using the ClustalW multiple alignment tool with the default parameters in MEGA 7.0. The phylogenetic analysis of *gltA* for SFGR, *16S rRNA* for *Anaplasma* spp., *16S rRNA* for *Coxiella* spp., or *18S rRNA* for *Babesia* spp. was performed using the maximum likelihood method based in MEGA 7.0. Bootstrap values were estimated for 1000 replicates [41, 42].

## Results

### Taxonomic classification

A total of 239 adult hard ticks were identified as *D. nuttalli* (*n* = 203) (accession number: MK213083.1) and *I. persulcatus* (*n* = 36) (accession number: MH790201.1) based on morphological identifications confirmed by species-specific PCR and sequencing assay. Ten pools of *D. nuttalli* DNA samples were finally analyzed by mNGS on the Illumina HiSeq platform. Sequencing yielded between 5970 and 7475 million reads per pool, while all were of high quality (Clean_Q20 > 96%) (shown in Additional file 1: Table S1). The construction of a metagenomic library of *I. persulcatus* DNA samples failed.

**Fig. 2:**
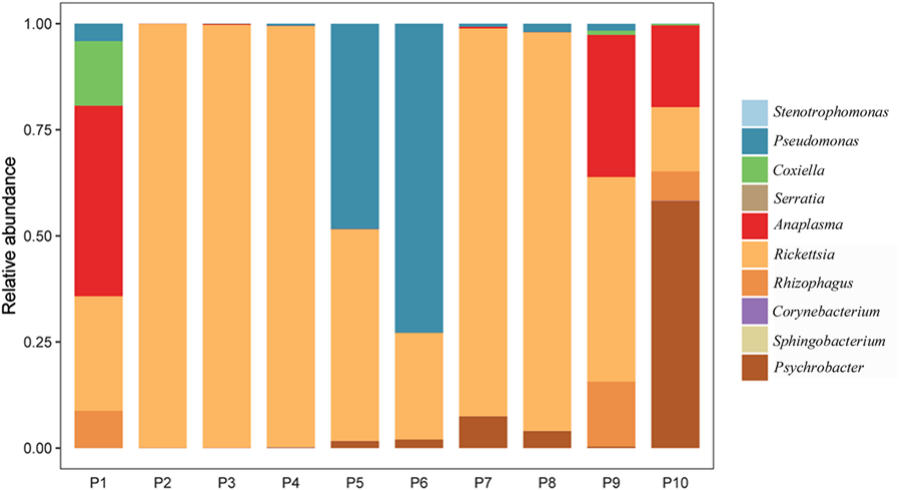
Relative abundances of potential top 10 pathogens at the genus level in pooled *D. nuttalli* samples. P1–P10, pooled DNA samples of *D. nuttalli* for metagenomic next-generation sequencing

The presence of the bacterial genera *Rickettsia*, *Anaplasma*, and *Coxiella* in the pooled tick samples was confirmed by the taxonomic profiles at genus level (Fig. 2; Table 2). *Rickettsia* spp. were most abundant in sample pools 2–4 and 7–8 and also abundant in other pools. In pools 1, 9, and 10, *Anaplasma* spp. were abundant. However, *Coxiella* spp. were abundant only in pool 1. In addition, *Pseudomonas* spp. were most abundant in pools 5 and 6, and *Psychrobacter* spp. were most abundant in pool 10 (Table 2).

**Table 2:** Number of genes matched to reference genomes in individual pooled DNA sample using mNGS

Taxonomy	Pooled tick DNA samples									
	P1	P2	P3	P4	P5	P6	P7	P8	P9	P10
*Pseudomonas*	61,323	0	9	81	8984	12,303	39	1079	305	29
*Stenotrophomonas*	11,392	2	0	1	38	11	1	5	4	0
*Sphingobacterium*	7157	0	0	0	0	0	0	0	0	0
*Serratia*	4748	0	1	1	2	1	1	4	2	0
*Corynebacterium*	100	2	3	13	7	2	1	4	2	62
*Rickettsia*	78	2002	1688	1922	1978	1968	1339	2026	2044	1647
*Psychrobacter*	2	6	0	12	504	953	220	880	140	1423
*Anaplasma*	157	5	6	5	7	5	4	5	416	158
*Coxiella*	54	0	0	0	0	0	0	0	81	66
*Rhizophagus*	29	1	2	2	1	2	0	2	111	71

### Prevalence of tick-borne pathogens in individual ticks

By mNGS, the important pathogenic bacterial genera *Rickettsia*, *Anaplasma*, and *Coxiella* were found in the pooled tick samples, and thus each tick was detected by the genus-/group-specific PCR combined with sequencing in order to identify the TBPs carried by it. In addition, *Babesia* spp. were often detected in ticks, and thus each tick was detected by *Babesia*-specific PCR.

As a result, *R. raoultii* (45.32%, 92/203), *Candidatus R. tarasevichiae* (5.42%, 11/203), *Anaplasma* sp. Mongolia (26.6%, 54/203), *Coxiella-*like endosymbiont (CLE) (53.69%, 109/203), and *Babesia venatorum* (7.88%, 16/203) were detected in *D. nuttalli*, while *R. raoultii* (30.56%, 11/36), *Anaplasma* sp. Mongolia (27.8%, 10/36), and CLE (27.8%, 10/36) were detected in *I. persulcatus* (Table 3).

**Table 3:** Prevalence of tick-borne pathogens in individual ticks

Pathogen	Number of individual ticks positive for single and co-infections	
	*D. nuttalli* (*n* = 203)	*I. persulcatus* (*n* = 36)
Single		
* Rickettsia raoultii*	22 (10.84%)	7 (19.44%)
* Candidatus R. tarasevichiae*	3 (1.48%)	-
* Anaplasma* sp. Mongolia	9 (4.43%)	8 (22.22%)
* Coxiella*-like endosymbiont	41 (20.20%)	5 (13.89%)
* Babesia venatorum*	8 (3.94%)	–
Double		
* R. raoultii, Anaplasma* sp. Mongolia	6 (2.96%)	–
* Candidatus R. tarasevichiae, Anaplasma* sp. Mongolia	5 (2.46%)	–
* Candidatus R. tarasevichiae, Coxiella*-like endosymbiont	1 (0.49%)	–
* R. raoultii, Coxiella*-like endosymbiont	29 (14.29%)	3 (8.33%)
* R. raoultii, B. venatorum*	2 (0.99%)	–
* Anaplasma* sp. Mongolia*, Coxiella*-like endosymbiont	4 (1.97%)	1 (2.78%)
Triple		
* R. raoultii, Anaplasma* sp. Mongolia*, Coxiella-like endosymbiont*	28 (13.79%)	1 (2.78%)
* Candidatus R. tarasevichiae, Anaplasma* sp. Mongolia, *Coxiella-like endosymbiont*	1 (0.49%)	–
* R. raoultii, Coxiella-like endosymbiont, B. venatorum*	5 (2.46%)	–
* Candidatus R. tarasevichiae, Anaplasma* sp. Mongolia*, B. venatorum*	1 (0.49%)	–
Total	165 (81.82%)	25 (69.44%)

### Co-infection in individual ticks

In 190 TBP-positive ticks, 87 ticks (45.79%) were found to be co-infected with more than one species identified in the present study (Table 3). The dual- and triple-pathogen/endosymbiont co-infections were detected in 40.39% of *D. nuttalli* and 13.89% of *I. persulcatus*. The dual co-infection with *R. raoultii* and CLE and the triple co-infection with *R. raoultii*, *Anaplasma* sp. Mongolia and CLE were most frequent in *D. nuttalli* (Table 3).

### Phylogenetic analysis

**Fig. 3:**
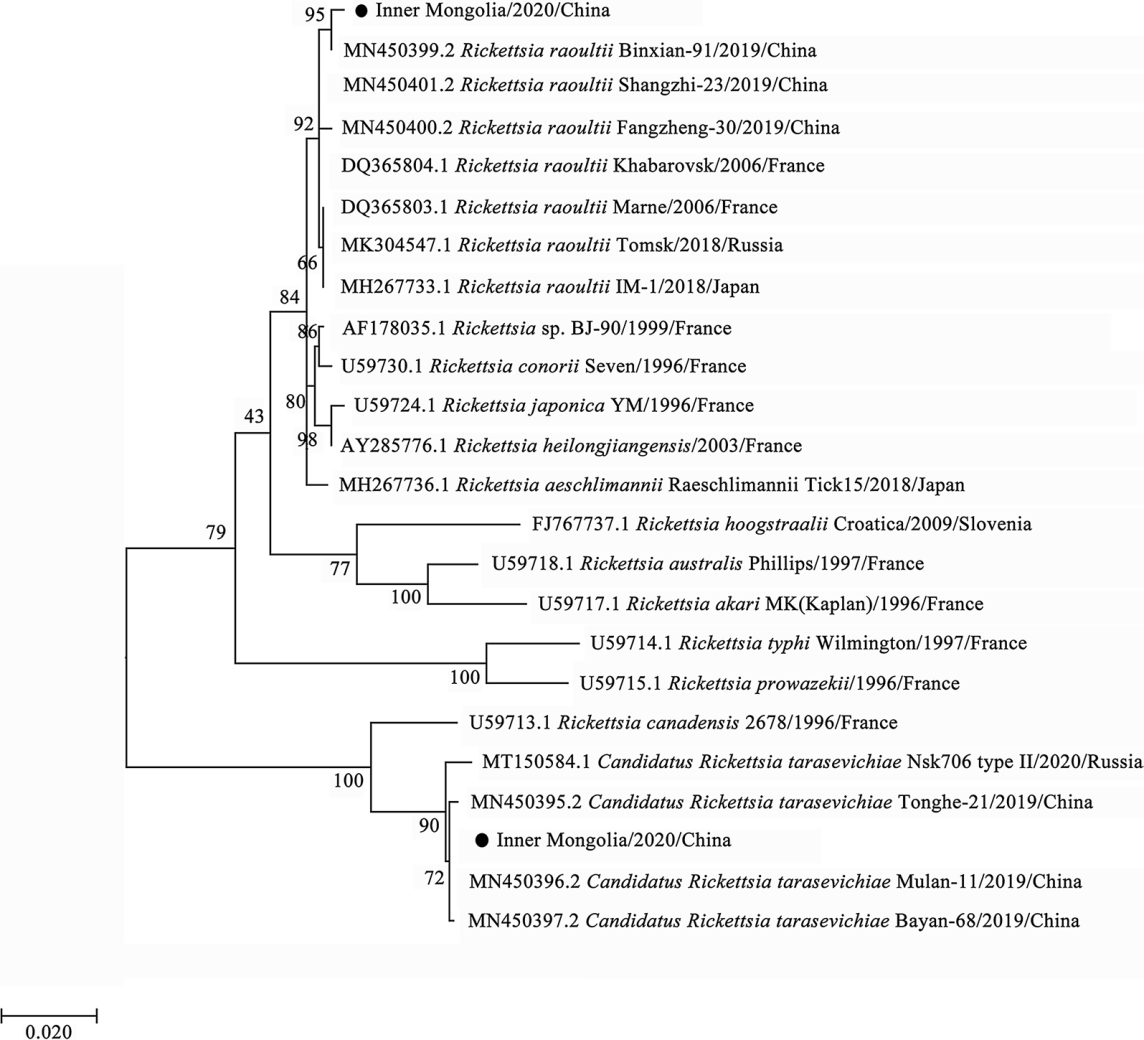
Phylogenetic tree of *R. raoultii* and *Candidatus R. tarasevichiae* in ticks based on partial *gltA* gene sequence similarity. The sequence obtained in this study is indicated with a black dot. Sequences were aligned using the MEGA 7 (version 7.0) software package. Phylogenetic analysis was performed by the maximum likelihood method, and bootstrap values were estimated for 1000 replicates

**Fig. 4:**
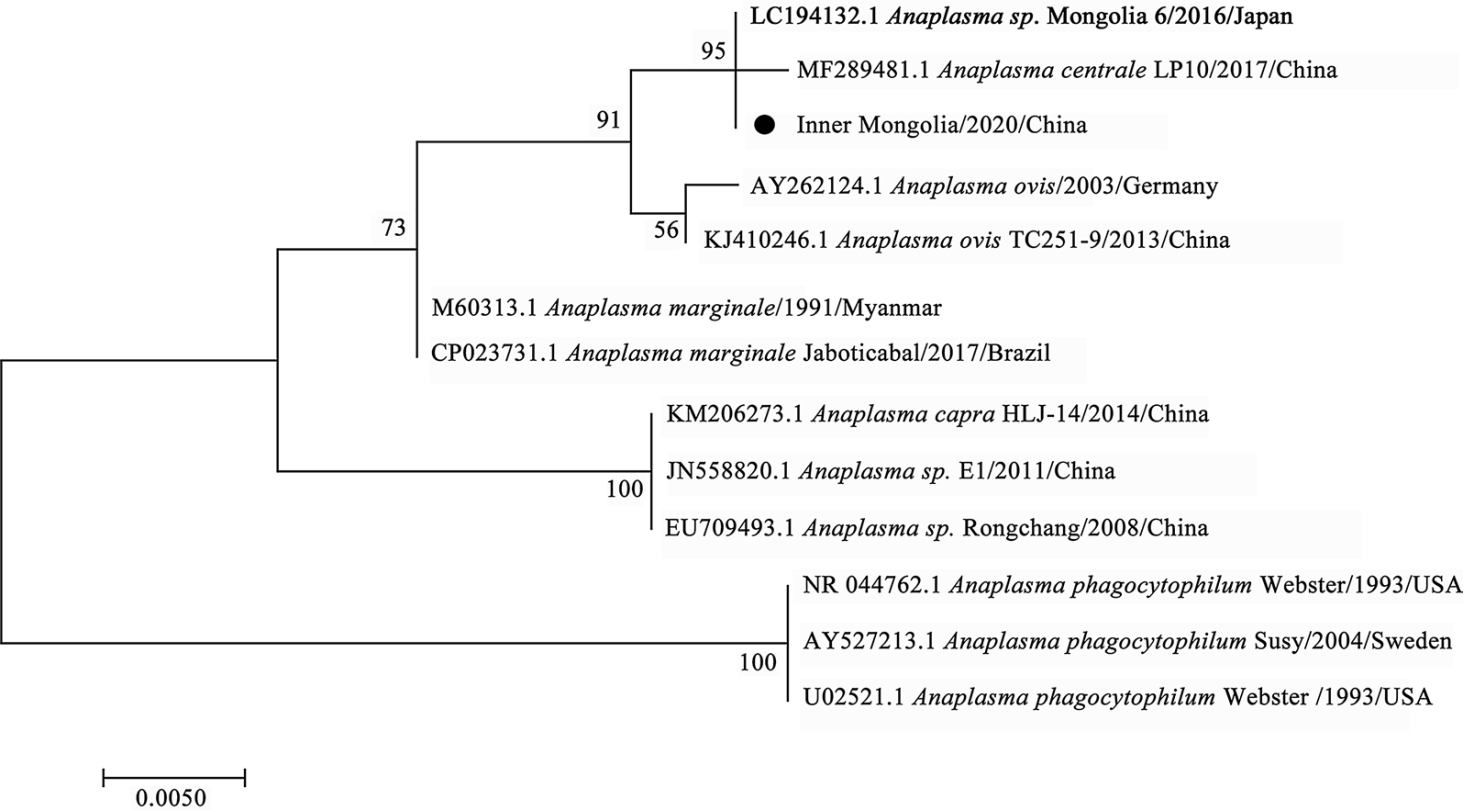
Phylogenetic tree of *Anaplasma* sp. Mongolia in ticks based on partial 16S *rRNA* gene sequence similarity. The sequence obtained in this study is indicated with a black dot. Sequences were aligned using the MEGA 7 (version 7.0) software package. Phylogenetic analysis was performed by the maximum likelihood method, and bootstrap values were estimated for 1000 replicates

**Fig. 5:**
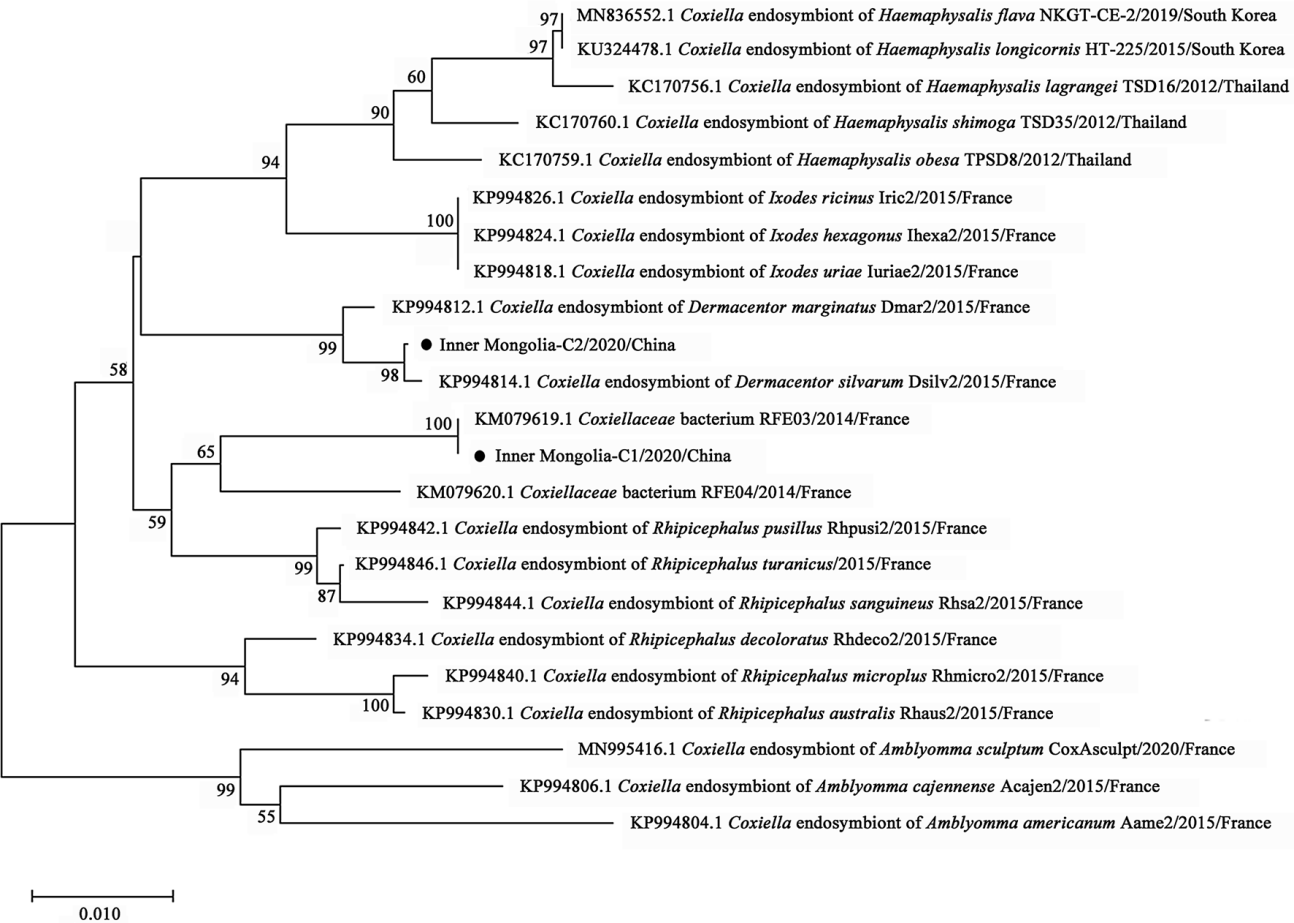
Phylogenetic tree of *Coxiella*-like endosymbionts in ticks based on partial 16S *rRNA* gene sequence similarity. The sequence obtained in this study is indicated with a black dot. Sequences were aligned using the MEGA 7 (version 7.0) software package. Phylogenetic analysis was performed by the maximum likelihood method, and bootstrap values were estimated for 1000 replicates

**Fig. 6:**
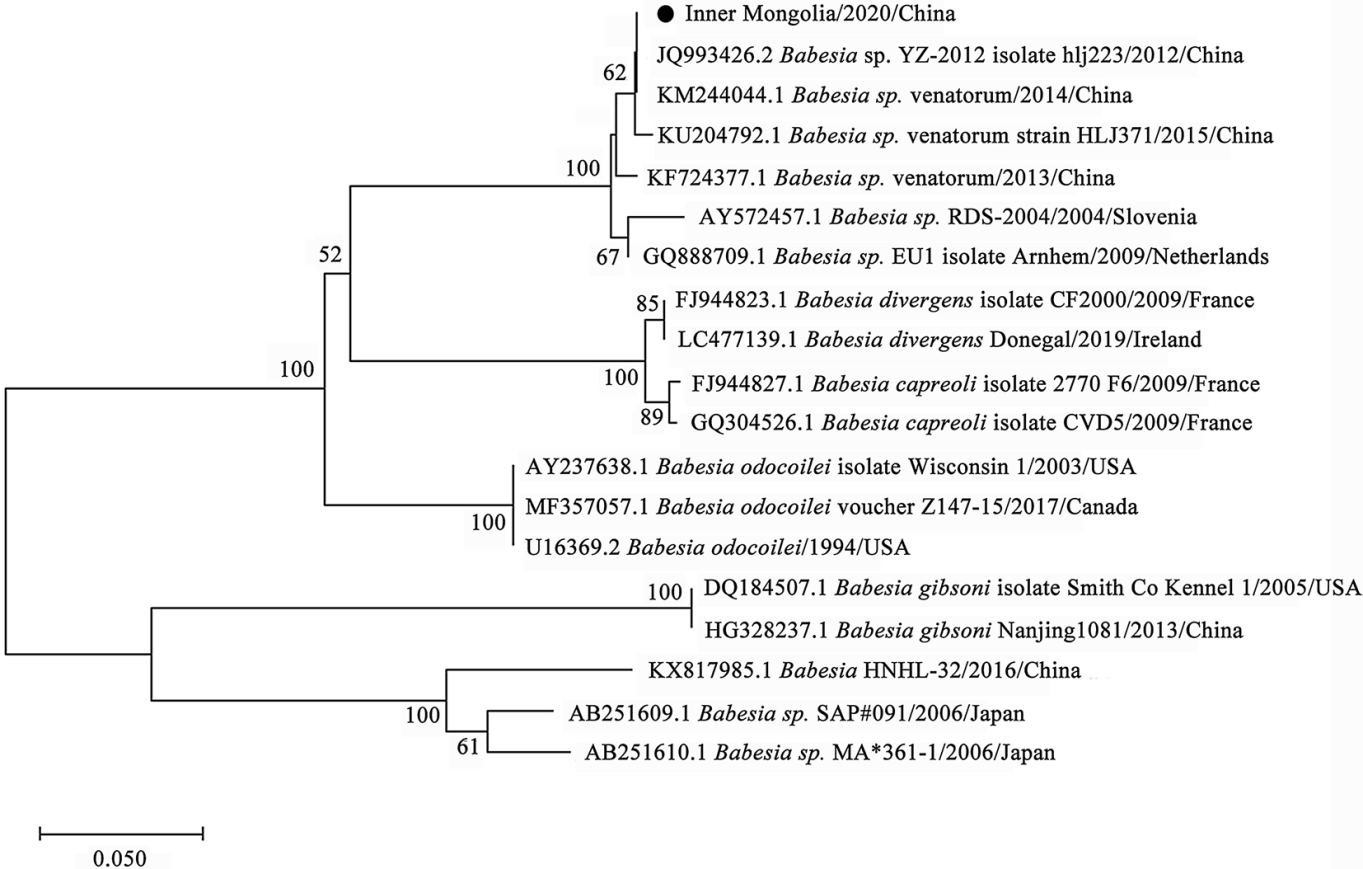
Phylogenetic tree of *B. venatorum* in ticks based on partial 18S *rRNA* gene sequence similarity. The sequence obtained in this study is indicated with a black dot. Sequences were aligned using the MEGA 7 (version 7.0) software package. Phylogenetic analysis was performed by the maximum likelihood method, and bootstrap values were estimated for 1000 replicates

By phylogenetic analysis, *R. raoultii* and *Candidatus R. tarasevichiae* were placed in a clade with *R. raoultii* Binxian-91 (MN450399.2) and *Candidatus R. tarasevichiae* (MN450396.2, MN450397.2), respectively (Fig. 3). *Anaplasma* sp. Mongolia identified in both *D. nuttalli* and *I. persulcatus* were shown to be clustered with *Anaplasma* sp. Mongolia 6 (LC194132.1) (Fig. 4). *Coxiella*-like endosymbiont identified in *D. silvarum* and in *I. persulcatus* were placed in a clade with *Coxiella* endosymbiont of *D. silvarum* (KP994814.1) and in a clade with *Coxiellaceae* bacterium RFE03 (KM079619.1), respectively (Fig. 5). *B*. *venatorum* was most close to *Babesia* sp. *Venatorum* strain HLJ371 (KU204792.1) and *Babesia* sp. YZ-2012 isolate hlj223 (JQ993426.2) (Fig. 6).

## Discussion

In recent years, much attention has been focused on ticks and TBPs in China. However, although a variety of pathogens have been identified, co-infection with multiple pathogens in hard ticks has rarely been investigated. In this study, we applied mNGS combined with nested PCR to survey TBPs in *D. nuttalli* and *I. persulcatus* feeding on cattle in Inner Mongolia, China.

By mNGS, the endosymbionts including *Coxiella* spp., *Rickettsia* spp., *Francisella* spp., and “*Candidatus* Midichloria mitochondrii” have been recognized as the most abundant bacterial species identified frequently in entirely homogenized ticks [43]. Qiu et al. applied NGS to examine the microbiomes of salivary glands of ticks collected in Japan, revealing a large number of bacterial genera, including 71 *I. ovatus*, 127 *I. persulcatus*, and 59 *H. flava*, and detecting some of the medically important bacteria including *Coxiella* spp., *Ehrlichia* spp., and *Rickettsia* spp. [44].

In the present study, the pooled DNA samples of *D. nuttalli* and *I. persulcatus* collected were assayed by mNGS. The result revealed the presence of the bacterial genera *Rickettsia*, *Anaplasma*, and *Coxiella* in these ticks. In order to identify the bacteria at species level, each tick was detected by SFGR-, *Anaplasma* and *Ehrlichia*-, and *Coxiella*-specific PCR as well as *Babesia*-specific PCR, respectively. After sequencing of the DNA fragments amplified by PCR and the sequence comparison, two *Rickettsia* species (*R. raoultii* and *Candidatus R. tarasevichiae*), one *Anaplasma* species (*Anaplasma* sp. Mongolia), *B*. *venatorum*, and CLEs were found in *D. nuttalli*, while *R. raoultii*, *Anaplasma* sp. Mongolia, and CLEs were also found in *I. persulcatus*.

*R. raoultii*, a species of SFGR, was firstly detected in *Dermacentor* ticks collected in Russia in 1999 and isolated from *Dermacentor* ticks and named in 2008 [45]. It is one of the causative agents of tick-borne lymphadenopathy (TIBOLA), which is also known as *Dermacentor*-borne necrosis erythema and lymphadenopathy (DEBONEL) in humans [46]. *R. raoultii* has been found to be present in various ticks, including *Dermacentor*, *Haemaphysalis*, *Rhipicephalus*, *Hyalomma*, and *Amblyomma*. In the present study, *R. raoultii* was detected in 45.32% of *D. nuttalli* and 30.56% of *I. persulcatus*, suggesting that it was the dominant *Rickettsia* species prevalent in the hard ticks in Inner Mongolia, and this may have health implications, as humans may suffer from spotted fever after experiencing a tick bite from this region. Phylogenetic analysis showed that the *R. raoultii* strains in *D. nuttalli* and *I. persulcatus* were identical and most related with *R. raoultii* Binxian-91 from *H. longicornis* in Shandong Province of China (MN450399.2), suggesting that its geographical distribution is wider in China.

*Candidatus R. tarasevichiae*, an emerging tick-borne pathogen, is also a species of SFGR. It was first detected in *I. persulcatus* collected from the southern Urals and Siberia in 2003 [47] and then found in *Haemaphysalis* ticks in Far East regions in Russia [48]. Human cases caused by *Candidatus R. tarasevichiae* have been found in China and Russia [49, 50]. In this study, *Candidatus R. tarasevichiae* was detected in 5.42% of *D. nuttalli*, which was most related to the *Candidatus R. tarasevichiae* Mulan-11 strain (MN450396.2) and Bayan-68 strain (MN450397.2) from *I. persulcatus* in China in phylogenetic analysis*.* This is the first time that *Candidatus R. tarasevichiae* has been detected in *D. nuttalli*.

*Anaplasma* sp. Mongolia was firstly detected in *D. nuttalli* [51] and bovine blood in Mongolia [51, 52], demonstrating that the *Anaplasma* species is an important cattle pathogen. In this study, *Anaplasma* sp. Mongolia was detected in 26.6% of *D. nuttalli* and in 27.8% of *I. persulcatus*. This study is thus the first in the world to report the presence of *Anaplasma* sp. Mongolia in *I. persulcatus*.

CLEs are relatively common in the microbiota of various tick species around the world, forming multiple subclusters in the cluster of the genus *Coxiella* in phylogenetic analysis [39, 53]. The presence of these symbiotic bacteria in ticks confers crucial and diverse benefits to the host, affecting its development, nutrition, chemical defense, or reproduction [53–55]. The prevalence of CLEs is from 6.25% in *Rhipicephalus sanguineus* to 100% in *Amblyomma americanum* in North America and Europe [53]. In the present study, CLEs were detected in 53.69% of *D. nuttalli* and 27.78% of *I. persulcatus*. Phylogenetic analysis suggested that the CLE strain of *D. nuttalli*, which was mostly related to that (KP994814.1) from *D. silvarum* in France, was different from that of *I. persulcatus*, which was mostly related to that (KM079619.1) of *Haemaphysalis concinna* from Russia.

*Babesia* spp. are the pathogenic agents of babesiosis in humans and animals*.* In the present study, *B. venatorum* was detected in 7.88% of *D. nuttall*. However, *Babesia* spp. was not found in the pooled tick samples by mNGS assay, which might be caused by the extremely low abundance of *Babesia* spp. in the pooled samples. According to phylogenetic analysis, *B. venatorum* of *D. nuttalli* was mostly related to *B. venatorum* strains YZ-2012 (JQ993426.2) and HLJ371 (KU204792.1) detected in *I. persulcatus* in Heilongjiang Province of China. *B. venatorum* can cause human infection masquerading as hemophagocytic syndrome [56].

In the present study, the multiple pathogen/endosymbiont co-infections were detected in 40.39% of *D. nuttalli* and 13.89% of *I. persulcatus*. Ticks may acquire multiple pathogenic species during blood feeding on their vertebrate hosts, and the hosts may also be infected by the pathogens carried by ticks. Due to the development of molecular diagnostic methods, more and more cases with co-infection of multiple TBPs have been reported [57]. The co-infection may be the result of a single tick bite by the tick carrying more than one pathogen or the result of multiple bites by ticks carrying different pathogens. Therefore, the prevalence of co-infection with TBPs in people living in the area close to the natural focus of TBPs should be investigated in the future.

## Conclusions

This study proves that various vertically transmitted pathogens co-inhabit *D. nuttalli* and *I. persulcatus*, and is the first to report that *Candidatus R. tarasevichiae* has been found in *D. nuttalli* in China, and the first in the world to report that *Anaplasma* sp. Mongolia has been detected in *I. persulcatus*. This study provides insight into the microbial diversity of *D. nuttalli* and *I. persulcatus* in Inner Mongolia, China, and indicates that cattle in Inner Mongolia are exposed to several TBPs.

## Supplementary Information

**Additional file 1**: **Table S1**. Pooling strategy for metagenomic next-generation sequencing.

## Data Availability

All data supporting the conclusions of this article are included in the article.

## Abbreviations

mNGS: Metagenomic next-generation sequencing
CLE: *Coxiella*-like endosymbiont
TBP: Tick-borne pathogen
PCR: Polymerase chain reaction
rRNA: Ribosomal RNA
PBS: Phosphate-buffered saline
COI: Mitochondrial cytochrome c oxidase I
NCBI: National Center for Biotechnology Information
BLAST: Basic Local Alignment Search Tool
SFGR: Spotted fever group *Rickettsia*
ORF: Open reading frame
LCA: Lowest common ancestor

## References

[1] Liu XY, Bonnet SI (2014). Hard tick factors implicated in pathogen transmission. PLoS Negl Trop Dis..

[2] Duan DY, Liu GH, Cheng TY (2020). Microbiome analysis of the saliva and midgut from partially or fully engorged female adult *Dermacentor silvarum* ticks in China. Exp Appl Acarol..

[3] Papa A, Tsioka K, Kontana A, Papadopoulos C, Giadinis N (2017). Bacterial pathogens and endosymbionts in ticks. Ticks Tick Borne Dis..

[4] Ahantarig A, Trinachartvanit W, Baimai V, Grubhoffer L (2013). Hard ticks and their bacterial endosymbionts (or would be pathogens). Folia Microbiol (Praha)..

[5] Li LH, Zhang Y, Wang JZ, Li XS, Yin SQ, Zhu D (2018). High genetic diversity in hard ticks from a China-Myanmar border county. Parasit Vectors..

[6] Sheng J, Jiang M, Yang M, Bo X, Zhao S, Zhang Y (2019). Tick distribution in border regions of Northwestern China. Ticks Tick Borne Dis..

[7] Fan MY, Wang JG, Jiang YX, Zong DG, Lenz B, Walker DH (1987). Isolation of a spotted fever group rickettsia from a patient and related ecologic investigations in Xinjiang Uygur Autonomous Region of China. J Clin Microbiol..

[8] Wu XB, Na RH, Wei SS, Zhu JS, Peng HJ (2013). Distribution of tick-borne diseases in China. Parasit Vectors..

[9] Zhang L, Liu Y, Ni D, Li Q, Yu Y, Yu XJ (2008). Nosocomial transmission of human granulocytic anaplasmosis in China. JAMA.

[10] Lu Z, Broker M, Liang G (2008). Tick-borne encephalitis in mainland China. Vector Borne Zoonotic Dis..

[11] Qi C, Zhou D, Liu J, Cheng Z, Zhang L, Wang L (2011). Detection of *Babesia* divergens using molecular methods in anemic patients in Shandong Province, China. Parasitol Res..

[12] Diuk-Wasser MA, Vannier E, Krause PJ (2016). Coinfection by *Ixodes* tick-borne pathogens: ecological, epidemiological, and clinical consequences. Trends Parasitol..

[13] Chisu V, Loi F, Foxi C, Chessa G, Masu G, Rolesu S (2020). Coexistence of tick-borne pathogens in ticks collected from their hosts in Sardinia: an update. Acta Parasitol..

[14] Cutler SJ, Vayssier-Taussat M, Estrada-Pena A, Potkonjak A, Mihalca AD, Zeller H (2021). Tick-borne diseases and co-infection: current considerations. Ticks Tick Borne Dis..

[15] Guo DH, Zhang Y, Fu X, Gao Y, Liu YT, Qiu JH (2016). Complete mitochondrial genomes of *Dermacentor silvarum* and comparative analyses with another hard tick *Dermacentor nitens*. Exp Parasitol..

[16] Batu N, Wang Y, Liu Z, Huang T, Bao W, He H (2020). Molecular epidemiology of *Rickettsia* sp. and *Coxiella burnetii* collected from *Hyalomma asiaticum* in Bactrian camels (*Camelus bactrianus*) in inner Mongolia of China. Ticks Tick Borne Dis..

[17] Yin X, Guo S, Ding C, Cao M, Kawabata H, Sato K (2018). Spotted fever group rickettsiae in Inner Mongolia, China, 2015–2016. Emerg Infect Dis..

[18] Feng J, Wu M, Huang T, Zhang J, Renbatu N (2019). Identification of two genotypes of *Argas persicus* and associated *rickettsia*-specific genes from different regions of Inner Mongolia. J Parasitol.

[19] Yin H, Luo J (2007). Ticks of small ruminants in China. Parasitol Res..

[20] Yu Z, Wang H, Wang T, Sun W, Yang X, Liu J (2015). Tick-borne pathogens and the vector potential of ticks in China. Parasit Vectors..

[21] Chitimia L, Lin RQ, Cosoroaba I, Wu XY, Song HQ, Yuan ZG (2010). Genetic characterization of ticks from southwestern Romania by sequences of mitochondrial *cox1* and *nad5* genes. Exp Appl Acarol..

[22] Karlsson FH, Tremaroli V, Nookaew I, Bergstrom G, Behre CJ, Fagerberg B (2013). Gut metagenome in European women with normal, impaired and diabetic glucose control. Nature.

[23] Karlsson FH, Fak F, Nookaew I, Tremaroli V, Fagerberg B, Petranovic D (2012). Symptomatic atherosclerosis is associated with an altered gut metagenome. Nat Commun..

[24] Luo R, Liu B, Xie Y, Li Z, Huang W, Yuan J (2012). SOAPdenovo2: an empirically improved memory-efficient short-read de novo assembler. Gigascience..

[25] Nielsen HB, Almeida M, Juncker AS, Rasmussen S, Li J, Sunagawa S (2014). Identification and assembly of genomes and genetic elements in complex metagenomic samples without using reference genomes. Nat Biotechnol..

[26] Li J, Jia H, Cai X, Zhong H, Feng Q, Sunagawa S (2014). An integrated catalog of reference genes in the human gut microbiome. Nat Biotechnol..

[27] Qin N, Yang F, Li A, Prifti E, Chen Y, Shao L (2014). Alterations of the human gut microbiome in liver cirrhosis. Nature.

[28] Li W, Godzik A (2006). Cd-hit: a fast program for clustering and comparing large sets of protein or nucleotide sequences. Bioinformatics.

[29] Fu L, Niu B, Zhu Z, Wu S, Li W (2012). CD-HIT: accelerated for clustering the next-generation sequencing data. Bioinformatics.

[30] Qin J, Li R, Raes J, Arumugam M, Burgdorf KS, Manichanh C (2010). A human gut microbial gene catalogue established by metagenomic sequencing. Nature.

[31] Villar E, Farrant GK, Follows M, Garczarek L, Speich S, Audic S (2015). Ocean plankton. Environmental characteristics of Agulhas rings affect interocean plankton transport. Science.

[32] Cotillard A, Kennedy SP, Kong LC, Prifti E, Pons N, Le Chatelier E (2013). Dietary intervention impact on gut microbial gene richness. Nature.

[33] Le Chatelier E, Nielsen T, Qin J, Prifti E, Hildebrand F, Falony G (2013). Richness of human gut microbiome correlates with metabolic markers. Nature.

[34] Buchfink B, Xie C, Huson DH (2015). Fast and sensitive protein alignment using DIAMOND. Nat Methods..

[35] Huson DH, Auch AF, Qi J, Schuster SC (2007). MEGAN analysis of metagenomic data. Genome Res..

[36] Feng Q, Liang S, Jia H, Stadlmayr A, Tang L, Lan Z (2015). Gut microbiome development along the colorectal adenoma-carcinoma sequence. Nat Commun..

[37] Cheng C, Fu W, Ju W, Yang L, Xu N, Wang YM (2016). Diversity of spotted fever group Rickettsia infection in hard ticks from Suifenhe, Chinese-Russian border. Ticks Tick Borne Dis..

[38] Qin XR, Han FJ, Luo LM, Zhao FM, Han HJ, Zhang ZT (2018). *Anaplasma* species detected in *Haemaphysalis longicornis* tick from China. Ticks Tick Borne Dis..

[39] Duron O, Noel V, McCoy KD, Bonazzi M, Sidi-Boumedine K, Morel O (2015). The Recent Evolution of a Maternally-Inherited Endosymbiont of Ticks Led to the Emergence of the Q Fever Pathogen, *Coxiella burnetii*. PLoS Pathog..

[40] Hamsikova Z, Kazimirova M, Harustiakova D, Mahrikova L, Slovak M, Berthova L (2016). *Babesia* spp. in ticks and wildlife in different habitat types of Slovakia. Parasit Vectors..

[41] Kumar S, Stecher G, Tamura K (2016). MEGA7: molecular evolutionary genetics analysis version 7.0 for bigger datasets. Mol Biol Evol..

[42] Hall BG (2013). Building phylogenetic trees from molecular data with MEGA. Mol Biol Evol..

[43] Greay TL, Gofton AW, Paparini A, Ryan UM, Oskam CL, Irwin PJ (2018). Recent insights into the tick microbiome gained through next-generation sequencing. Parasit Vectors..

[44] Qiu Y, Nakao R, Ohnuma A, Kawamori F, Sugimoto C (2014). Microbial population analysis of the salivary glands of ticks; a possible strategy for the surveillance of bacterial pathogens. PLoS ONE.

[45] Mediannikov O, Matsumoto K, Samoylenko I, Drancourt M, Roux V, Rydkina E (2008). *Rickettsia raoultii* sp. Nov., a spotted fever group rickettsia associated with *Dermacentor* ticks in Europe and Russia. Int J Syst Evol Microbiol..

[46] Wijnveld M, Schotta AM, Pinter A, Stockinger H, Stanek G (2016). Novel *Rickettsia raoultii* strain isolated and propagated from Austrian *Dermacentor reticulatus* ticks. Parasit Vectors..

[47] Yuan TT, Ma L, Jiang BG, Fu WM, Sun Y, Jia N (2020). First Confirmed Infection Of *Candidatus Rickettsia Tarasevichiae* in rodents collected from Northeastern China. Vector Borne Zoonotic Dis..

[48] Eremeeva ME, Oliveira A, Moriarity J, Robinson JB, Tokarevich NK, Antyukova LP (2007). Detection and identification of bacterial agents in *Ixodes persulcatus* Schulze ticks from the north western region of Russia. Vector Borne Zoonotic Dis..

[49] Rudakov N, Samoylenko I, Shtrek S, Igolkina Y, Rar V, Zhirakovskaia E (2019). A fatal case of tick-borne rickettsiosis caused by mixed Rickettsia sibirica subsp. sibirica and *Candidatus Rickettsia tarasevichiae* infection in Russia. Ticks Tick Borne Dis..

[50] Jia N, Zheng YC, Jiang JF, Ma L, Cao WC (2013). Human infection with *Candidatus Rickettsia tarasevichiae*. N Engl J Med..

[51] Fischer T, Myalkhaa M, Krucken J, Battsetseg G, Batsukh Z, Baumann MPO (2020). Molecular detection of tick-borne pathogens in bovine blood and ticks from Khentii, Mangolia. Transbound Emerg Dis..

[52] Moore TC, Pulscher LA, Caddell L, von Fricken ME, Anderson BD, Gonchigoo B (2018). Evidence for transovarial transmission of tick-borne rickettsiae circulating in Northern Mongolia. PLoS Negl Trop Dis..

[53] Zhong J (2012). *Coxiella*-like endosymbionts. Adv Exp Med Biol..

[54] Ben-Yosef M, Rot A, Mahagna M, Kapri E, Behar A, Gottlieb Y (2020). *Coxiella*-like endosymbiont of *Rhipicephalus sanguineus* is required for physiological processes during ontogeny. Front Microbiol..

[55] Smith TA, Driscoll T, Gillespie JJ, Raghavan R (2015). A *Coxiella*-like endosymbiont is a potential vitamin source for the Lone Star tick. Genome Biol Evol..

[56] Blackberg J, Lazarevic VL, Hunfeld KP, Persson KEM (2018). Low-virulent *Babesia venatorum* infection masquerading as hemophagocytic syndrome. Ann Hematol..

[57] Moniuszko A, Dunaj J, Swiecicka I, Zambrowski G, Chmielewska-Badora J, Zukiewicz-Sobczak W (2014). Co-infections with *Borrelia* species, *Anaplasma phagocytophilum* and *Babesia* spp. in patients with tick-borne encephalitis. Eur J Clin Microbiol Infect Dis..

